# Climate change decouples dominant tree species in African savannas

**DOI:** 10.1038/s41598-023-34550-9

**Published:** 2023-05-10

**Authors:** Fezile P. Mtsetfwa, Laurence Kruger, Robert A. McCleery

**Affiliations:** 1 Department of Wildlife Ecology and Conservation, School of Natural Resource and Environment, University of Florida, Gainesville, FL USA; 2 School of Animal Plant and Environmental Sciences, University of the Witwatersrand, Johannesburg, South Africa; 3 Organisation for Tropical Studies, Skukuza, South Africa; 4 Biology Department, University of Cape Town, Cape Town, South Africa

**Keywords:** Ecology, Climate-change ecology, Tropical ecology

## Abstract

To understand how two dominant African savanna trees will continue to respond to climate changes, we examined their regeneration niche and adult tree distributions. Specifically, we wanted to (1) determine if distributional patterns were shifting, (2) predict future distributions under different climate change scenarios and (3) evaluate the realism of predicted future distributions. We randomly placed 40 grids into 6 strata across a climate gradient in the kingdom of Eswatini. Within these grids, we sampled adult and seedling marula (*Scelerocarya birrea*) and knobthorn (*Senegalia nigrecens*) trees and used the data to model their abundance. Next, we quantified shifts in distributional patterns (e.g., expansion or contraction) by measuring the current and projected areas of overlap between seedling and adult trees. Finally, we predicted future distributions of abundance based on predicted climate conditions. We found knobthorn seedlings within a small portion of the adult distribution, suggesting it was unlikely to track climate changes. Alternatively, finding marula seedlings on and beyond one edge of the adult distribution, suggested its range would shift toward cooler climates. Predicted future distributions suggest suitable climate for both species would transition out of savannas and into grasslands. Future projections (2041–2070) appeared consistent with observed distributions of marula, but knobthorn predictions were unrealistic given the lack of evidence for regeneration outside of its current range. The idiosyncratic responses of these species to climate change are likely to decouple these keystone structures in the coming decades and are likely to have considerable cascading effects including the potential rearrangement of faunal communities.

## Introduction

Changes to our planet’s climate are projected to accelerate in the coming decades^1^ with rates of change varying among the planet’s terrestrial systems^2,3^. Plant communities which are the defining features of most terrestrial systems^4,5^ are often characterized by their dominant species. In the face of a rapidly changing climate, these dominant plant species can respond by shifting their distribution to match climate conditions, die off, or adapt to the new conditions^6–8^. However, it is unlikely that the responses of individual species to climate change will not be uniform^9,10^, reshaping terrestrial biomes and creating no-analog communities with novel biotic interactions^11^.

Compared with other terrestrial biomes, tropical savannas, face heightened risks from a rapidly changing climate (moving at 0.6–1.26 km/year)^3^. Savannas are characterized by the co-dominance of trees and grasses^12^ and trees in this system struggle to tract rapid environmental changes because they are long lived (living up to 1000 yrs^13^). Additionally, many savanna trees are dependent on an increasingly depauperate mammal community^14,15^ for the long-distance dispersals necessary for tracking climate change^6^. Specifically, the defaunation of birds and large mammals has reduced the ability for plants to track climate change by ~ 60%^15^. Despite these threats, we still have very little understanding of the factors that determine the distributions of savanna trees under current conditions^16,17^ and little understanding of the rates of migration that trees will need to keep up with a moving climate^6,18,19^.

Most of our understanding of trees ability to respond to a changing climate comes from dynamic vegetation models (DVMs)^20–23^ and Species Distribution Models (SDMs)^24^. These approaches have both been useful in predicting biome scale responses to climate change but DVMs can overlook individual species responses^25^ and SDMs can be limited by the assumption that observed species distributions are in equilibrium with climate variables^22,26,27^. This assumption is particularly problematic for long-lived large tree species that were likely established under different climatic conditions. An alternative approach to using SDM and DVMs is to examine tree populations demographics and size class distributions across an environmental gradient^28^. In particular, understanding a species regeneration niche (conditions in the early life cycle e.g., seedling, sapling of a plant^29,30^) across climate gradients can help indicate future responses to climate change and help validate predictions from SDMs^31,32^.

Additionally, studying multiple size classes of trees aids in understanding the potentially different responses of important life stages^33,34^. Specifically, seedlings (i.e., juvenile size class distributions) can be used to project future plant distributions^34^. For example, the occurrence of seedlings in previously cooler and uncolonized areas, is indicative of trees that are tracking climate change^35^.

In this study, we wanted to understand the response of two different and dominant savanna trees to a changing climate. Our specific objectives were to determine (1) if differences in climatic suitability of seedling and adult trees suggested shifting distributional patterns (e.g., expansion or contraction), (2) how the trees’ climatic suitability would shape future distributional patterns under predicted climate change scenarios and (3) if predicted future distributions are realistic based on current regeneration patterns. Working across a climate gradient in the Kingdom of Eswatini, we predicted that one species (knobthorn, *Senegalia nigrecens*), would show resilience with both seedlings and adults widely distributed within its range to hotter and dryer conditions because of its high tolerance for heat and germination requirements^36,37^ but with little ability to track climate changes because of their limited dispersal ability^38^. In contrast, we predicted the other species (marula, *Scelerocarya birrea*) would be less resilient (areas without seedling within the adult range) but show evidence of tracking climate change because of human mediated dispersal mechanisms^39^. Finally, we predicted that projected future climate conditions will show a decoupling of the species distributions that manifests from the variation in their current regeneration niches.

## Materials and methods

### Site description

**Figure 1:**
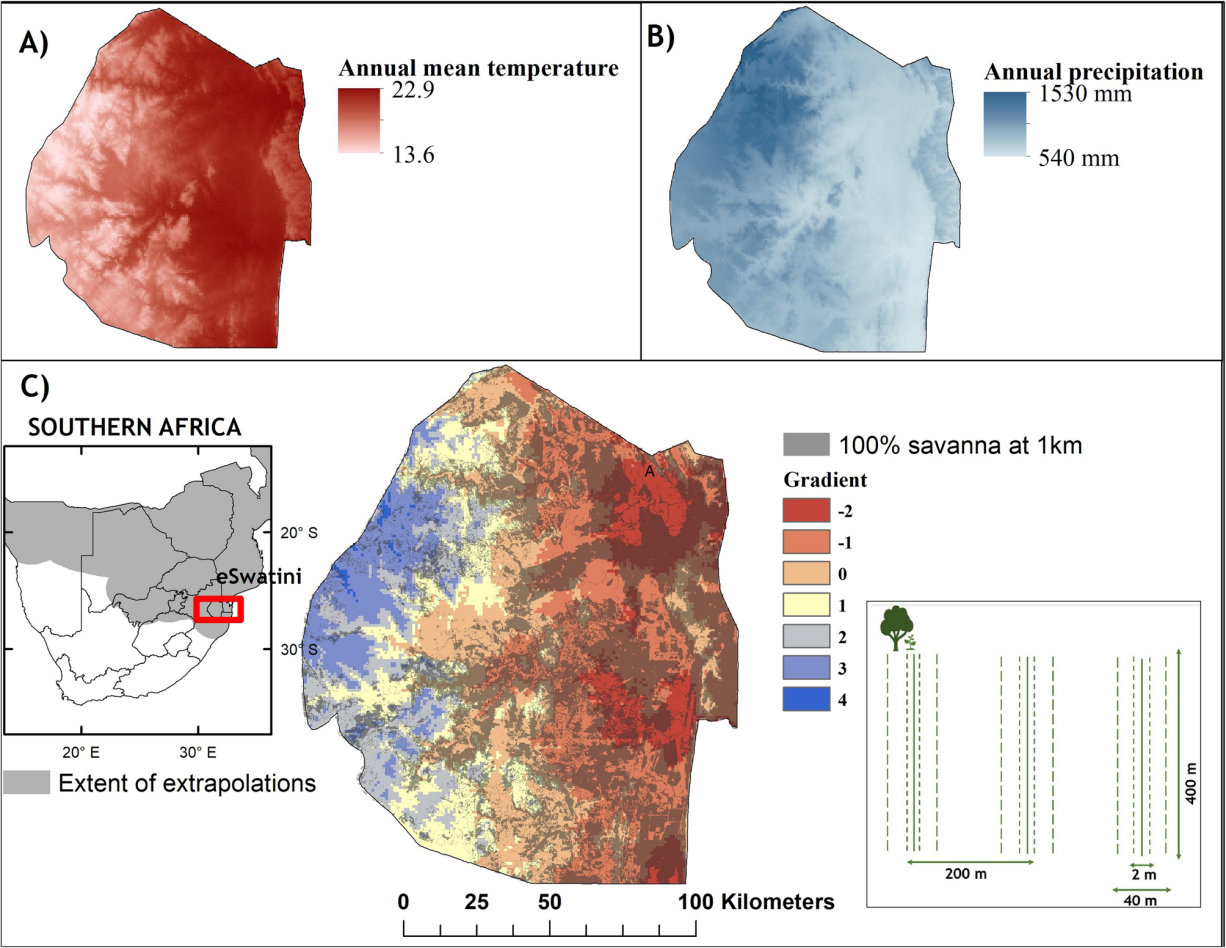
Map of study area in Eswatini. Panel (**A**) and (**B**) show the ranges of mean annual temperature (bioclim 01) and annual precipitation (bioclim 12). Panel (**C**), shows location of Eswatini and set up of the gridded transects across the climate gradient of Eswatini, grey areas on the map of Eswatini represent potential sampling sites with 100% savanna vegetaion at 1 km. This figure was produce using ArcMap 10.8.2 (Redlands, CA: Environmental Systems Research Institute).

Southern Africa is expected to see drastic changes in precipitation and temperatures in the coming decades, with a projected decrease of 1–3 mm and an increase 1–2 °C per 50 years respectively^40^. These predicted climate changes maybe even more pronounced for the extent of our study area in the kingdom of Eswatini (formerly Swaziland), a small landlocked country in southern Africa covering approximately 17,360 km^2^. The annual mean temperature of Eswatini is expected to increase by averages of 2.3 °C and 4.4 °C accompanied by a 3.4% and 8.7% decline in annual precipitation by the years 2050 and 2080 respectively under unmitigated emission scenarios (RCP 8.5)^41^. Although small, the country has a diverse altitude ranging from 100 m above sea level (a.b.l.) in the Lowveld to 1850 m a.b.l. in the high elevation grasslands i.e., Highveld of the country^42^. This altitudinal gradient is correlated with rainfall and temperature gradients, increasing from east to west and form the south towards the north–west (Fig. 1B). These gradients create considerable variation in Eswatini’s vegetation communities. Savanna communities occur in the east of the country, between altitudes of 200–900 m (Fig. 1C). In addition to climate, the dominant savanna vegetation in each region is moderated by terrain and soil properties along the altitudinal gradient^41^. The climate gradient in Eswatini makes for an ideal system for understanding how climate alters vegetation composition transitions in savannas across landscapes.

#### Tree species selection

We studied the response of knobthron and marula to climate change. These are widely occurring and dominant southern African tree species that are critical to the functioning of lowland savannas^43^. Like other dominant, long-lived, and scattered trees in African savannas, they are the defining architectural structures in the system, are wildly utilized by wildlife and people a like, and closely linked to biodiversity^44–47^. Among its many ecological functions, knobthorn is a staple food source for ungulate browsers^48^, specifically; kudu (*Tragelaphus strepsiceros*), steenbok (*Raphicerus campestris*), impala (*Aepyceros melampus*) and elephant (*Loxodonta africana*) that can strip and toppled them^49^. Knobthorn is also economically important for wood-based products and a source of fuel^50^. Marula is utilized by elephants and other medium bodied mammals^45,51^ and widely harvested for its fruit^39^.

Knobthorns flower at the beginning of the rainy season and fruit towards the end of the season, sometimes twice a year^52^. Knobthorn is self-dispersed, with seeds dropping directly below the plant or a few meters away, resulting in aggregations or clumps of trees^38^. Marula reaches reproductive maturity at an average of 14 cm stem diameter^14,39^. Marula has various mammal dispersers including elephants, vervet monkeys (*Cercopithecus aethiops*), chacma baboons (*Papio cynocephalus ursinus*)^53^, squirrels (*Xerus inauris*)^54^, and humans^39^. Marula therefore has a relatively longer dispersal range compared to the self-dispersed knobthorn. Increasingly, there is evidence that long distance dispersal has been from humans, particularly in systems with no elephant populations.

#### Climatic data and climate gradient

To determine how past climatic suitability has shaped current tree distributions and regeneration patterns, we obtained 20 climate variables and a layer for the altitude for Eswatini from the Worldclim-Global Climate Database (https://www.worldclim.org/bioclim). We downloaded data at the highest spatial resolution (~ 1 km). These bioclimatic variables were derived metrics averaged between 1950 and 2000. To determine which variables to use in our models, we considered biological significance and collinearity. We considered variables representing the lowest, highest and average temperature and precipitation metrics because they can influence the growth of young trees (i.e., seedlings) and the persistence of adults^52,55,56^. Additionally, to capture the interactive effects of temperature and precipitation, we considered potential evapotranspiration (PET) from the AFRICLIM 3.0.

To determine how the trees’ climatic suitability would shape their future distributional patterns, we predicted the distribution of their abundance under future climate conditions, using the AFRICLIM 3.0 data set. This is a regional climate model (RCM) nested within a general circulation model (GCM), downscaled to 1 km resolution. The regional model was based on the Worldclim v1.4 (1950–2000) contemporary baseline and projected under two representative concentration pathways, IPCC-AR5 (RCP4.5 and RCP8.5) to represent mid-century (2041–2070) and late-century (2071–2100) means^57^. To capture a climate gradient of both temperature and precipitation and select sampling strata, we performed a Principal Components Analysis (PCA) analysis on the Annual mean temperature and Annual precipitation using the package ‘RStoolbox’^58^. We selected the rasterstack of PCA 1 which accounted for most of the variation (> 70%) to use as our climate gradient. The resultant rasterstack provided a continuous (− 2.9 to 4) variable describing the temperature/precipitation gradient of Eswatini. To enable sampling across the gradient, we created discrete categories from the continuous range of values at intervals of 1, resulting in seven categories (Fig. 1), six of which were large enough to fit 2.5 km^2^ sampling grids (− 2.9 to 3).

To control for the potential deleterious effects of intensive land use change (i.e., urban development and agriculture land uses that preclude regeneration), we sampled within areas where trees have the best opportunity to track climate change, relatively undisturbed savannas. We identified different land covers based on a Landsat Enhanced Thematic Map of remotely sensed imagery at a 30 m × 30 m resolution developed by Eswatini National Trust Commission (ENTC) in 2017. To identify areas suitable for sampling we used a moving window analysis (used to calculate a value for a specific neighborhood of cells in a given raster) in the program R^59^, to select for areas with a 100% savanna cover at a focal radius of 1 km.

#### Tree surveys

Acknowledging that tree distributions are patchy^17^, we attempted to capture local variation by sampling grids with 3 transacts of 400 m each, transects were spaced 200 m apart (Fig. 1C). We randomly placed 6–8 grids in areas of savannas in each of the 6 climate strata defined above. Additionally, we placed grids > 10 km apart to help insure spatial independence. Overall, we surveyed marula and knobthorn trees on 120 × 400 m transects (48.0 km) within 40 grids across Eswatini.

We sampled trees visually using transect lines, a proven survey method for effectively sampling woody vegetation^50,56^. To maintain the assumption of equal detection among sites, the same observer slowly walked transect lines, surveying trees with a height < 1.0 m within 1 m on each side of the transect. Similarly, we sampled large trees > 1 m within 20 m of either side of the transect line^14^. We adopted this approach because we could consistently detect trees of the two size classes at these distances in both open and closed savannas. We corrected for number of individuals detected in the < 1 m category by multiplying the number of individuals observed in the sampled 2 m transect strip by 20. This ensured comparison with the larger trees (> 1 m) which were sampled across a 40 m transect strip. For each individual we encountered, we measured height (m), stem diameter at breast height (DBH) for large trees ≥ 1.3 m and basal stem diameter for seedlings^60^. Tree height was estimated using a 4 m long height measurement pole. We did not collect plant material in this study and were compliant with the IUCN Policy Statement on Research Involving Species at Risk of Extinction and the Convention on the Trade in Endangered Species of Wild Fauna and Flora.

#### Size-class definitions

To determine variation in how different life stages of marula and knobthorn corresponded with climate variation, we broadly classified individuals into adults and ‘young’ trees, which we refer to as seedlings from here on. We classified individual trees with height ≥ 3 m and (diameter at breast height) DBH ≥ 15 cm as adults, we categorized all other trees as ‘young’ trees. In making this classification we considered the major factors which can prevent large savanna trees recruiting into older size classes; reproductive maturity, fire resistance, and resistance to herbivory^36^. Reproductive maturity in marula can be observed in individuals with stem sizes as small as 7 cm in areas that go with long periods without fire, however, on average this value is ~ 14 cm^14^. Furthermore, savanna trees are most likely to survive fires once they reach a height of ≥ 3 m, particularly if there are longer intervals between fires^16,33^.

#### Statistical analyses

To determine if differences in climatic suitability of seedling and adult trees suggested shifting distributional patterns (e.g., expansion or contraction), first we measured the abundance (counts of individuals per category and transect) of marula and knobthorn seedlings and adults across a gradient of temperature and precipitation. To understand the climatic conditions that were influencing the abundance of both seedling and adults of both species^61,62^, we evaluated a suite of a priori models comprised of six variables (Mean Annual Temperature, Maximum Temperature of warmest month, Minimum Temperature of coldest month, Annual Precipitation, Precipitation of the wettest month and PET, see Supplementary Table S1 and S2 online). We evaluated these models of abundance using generalized linear-mixed models (GLMMs) with grid as a random variable and a zero-inflated negative binomial distribution using the package ‘glmmTMB’^63^ in the program R^59^. Count data representing the abundance of trees and seedlings is commonly modeled with GLMMs fit to either Poisson or negative binomial distributions depending on the amount of zero inflation^64–66^. Due to considerable correlation among our variables (R = 0.6 − 1.0) we generated and compared single variable models to avoid issue with model interpretation and overfitting. However, we added quadratic terms (x + x^2^) for each of these variables to account for the potential for non-linear responses of abundances across the gradient.

We compare the parsimony of our models using delta Akaike Information Criterion (∆AIC) calculated with the ‘bblme’ package^67^. We considered models within 2 AIC units of each other to be competing. We examined the relevance of parameters in the competing models using the *p*-value at < 0.05 (Wald test) and if their Confidence Interval (CI) included 0. We further examined the magnitude of relevant variables by plotting their predicted responses. Additionally, we examined model fit by extracting R^2^ values using the ‘sjstats’ package^68^. We predicted the expected abundance of trees across a continuous range of the best predictor variable using the “predict” function in the R package ‘glmmTMB’^63^. We report ‘optimal’ values for our best predictor variables as the values where maximum abundance of trees was predicted. We used spatial correlograms with non-parametric bootstrapping^69^ to investigate the residuals of our best fitting models^70^ and found no evicdence of spatial dependence.

To visualize how the abundances of marula and knobthorn adults and seedlings were distributed across the study area, we used the “predict” function in the R package ‘raster’^71^ to project abundance predictions onto the spatial extent of Eswatini. To determine the current amount and overlap of geographic space for seedling and adult trees, we converted the predicted abundance of each size class to a binary variable (0/1) corresponding to presence/ absence and plotted it. We then subtracted the distribution of adults from that of seedlings to get a distribution of areas that were previously suitable for seedling establishment but where no seedlings were modeled. This allowed us to understand how distributional patterns (e.g., expansion or contraction) based on climate suitability varied between seedlings and adults. Additionally, we estimated the area covered by these distributional patterns by calculating the number of pixels covered by each^72^.

Next, to determine how the trees’ climatic suitability would shape their future distributional patterns, we predicted their distributions under future climate conditions, using the AFRICLIM 3.0 data set. We projected future distribution for both species for our study area Eswatini and Southern Africa. We extrapolated the results to Southern African savannas^73^ in order to determine how much climate space would be lost at a regional scale, assuming observed local trends persisted across the region. Finally, we evaluated whether these future distributions aligned with our regeneration niche findings. Specifically, we wanted to determine if future predicted distributions were realistic given evidence of expansion or contraction of the regeneration niche.

## Results

We detected a total of 337 marula on fourteen of forty grids and 406 knobthorn trees on eight of forty grids. Eighty-four percent (n = 281) of recorded marula individuals were adults and only 16% were seedlings (n = 56). We recorded very few marula seedlings on average per grid, 75% of seedlings (n = 42) were aggregated on 2 of the 14 grids with detections. Of all the recorded knobthorn, 72% (n = 293) were adult trees and 28% (n = 113) were seedlings.

### Climate suitability

Climate suitability, as measured by total abundance and the abundance of adults and seedlings for both marula and knobthorn, was best described by models with quadratic temperature variables. Specifically, we found squared-annual mean temperature was the best predictor for knobthorn and the best predictors for marula were the squared variables annual mean temperature, maximum temperature of warmest month and PET (see Supplementary Table S3 online).

**Figure 2:**
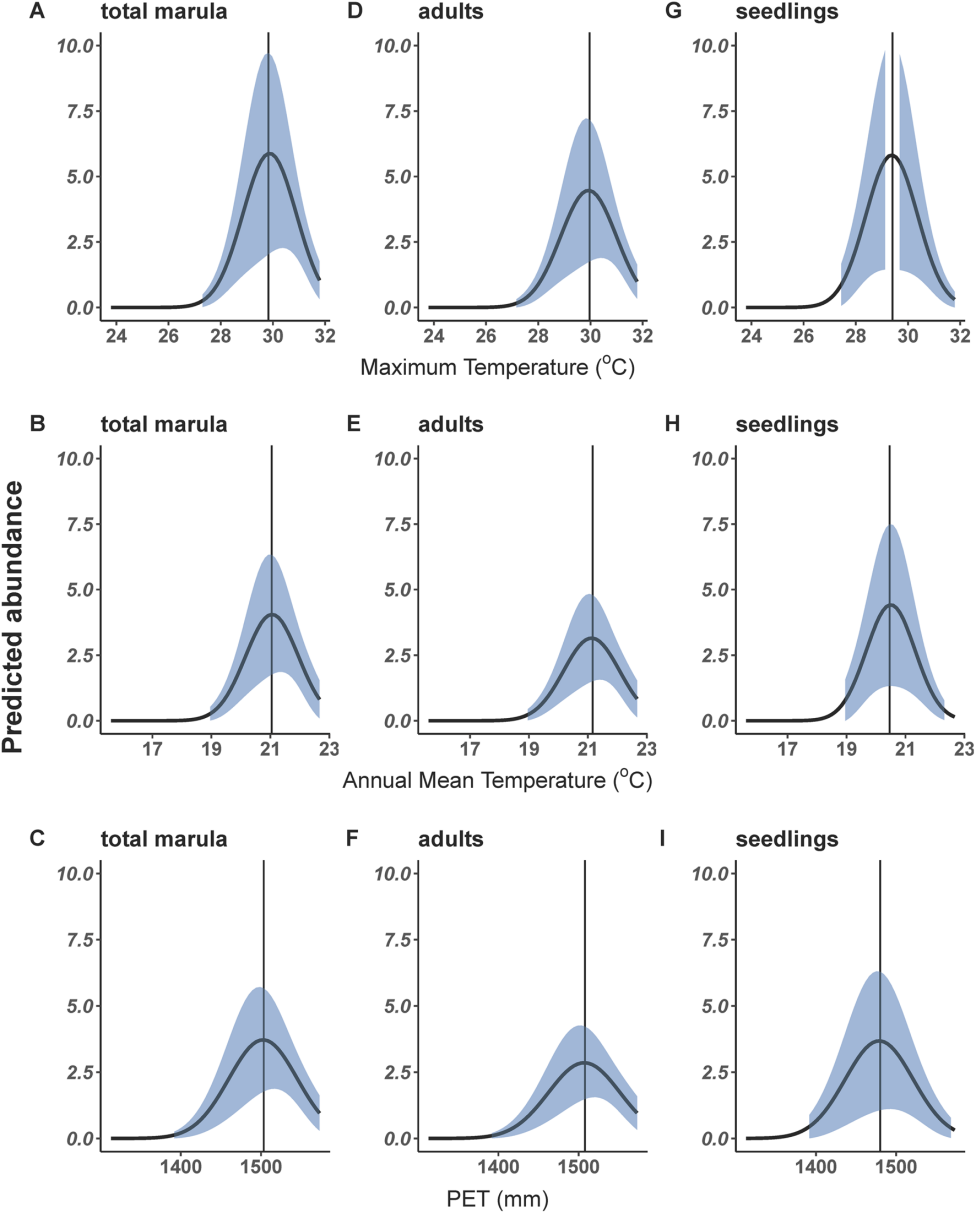
Predicted average abundances per grid of adults, seedlings and total marula across a gradient of maximum temperature of warmest month (**A**, **D**, **G**) and mean annual temperature (**B**, **E**, **H**) in  °C and PET (**C**, **F**, **I**). Vertical lines indicate optimal climate conditions (temperature/PET) for highest predicted abundance and the shaded region is the 95% CI.

The best predictive climate suitability models for the abundance of total marula was squared maximum temperature of the warmest month. Abundance of total marula increased with maximum temperature of the warmest month up to an optimal temperature of 29.8 °C, beyond which we observed decreases in abundance (Fig. 2A). The models for squared annual mean temperature (Fig. 2B, E and H) and PET (Fig. 2C, F and I) were competing models in explaining total abundance of marula trees (ΔAIC = 0.6) with optimal values at 21.0 °C and 1503.40 mm respectively (Fig. 2B and C).

Similarly, the best predictive climate suitability models for adult and seedling marula tree abundances were the squared variables of maximum temperature of the warmest month, optimal at 29.96 °C and 29.4 °C respectively (Fig. 2D and G). Additionally, PET^2^ was a competing model for predicting marula seedling abundances, with optimal values recorded at 1480.14 mm (Fig. 2I). The quadratic models for annual mean temperature and PET were also competing models in predicting marula adult abundances. The highest abundances of adult marula trees were recorded at 21.16 °C and 1508.00 mm (Fig. 2H and F).

The climate suitability of knobthorn, as measured by the total abundance, adult abundance and seedling abundance was best described by a quadratic model for annual mean temperature and there were no competing models. The highest abundances were recorded at optimal temperatures of 22.09 °C, 22.09 °C and 22.18 °C annual mean temperature for total abundance, adult and seedling abundances respectively (see Supplementary Fig. S1 online).

**Figure 3:**
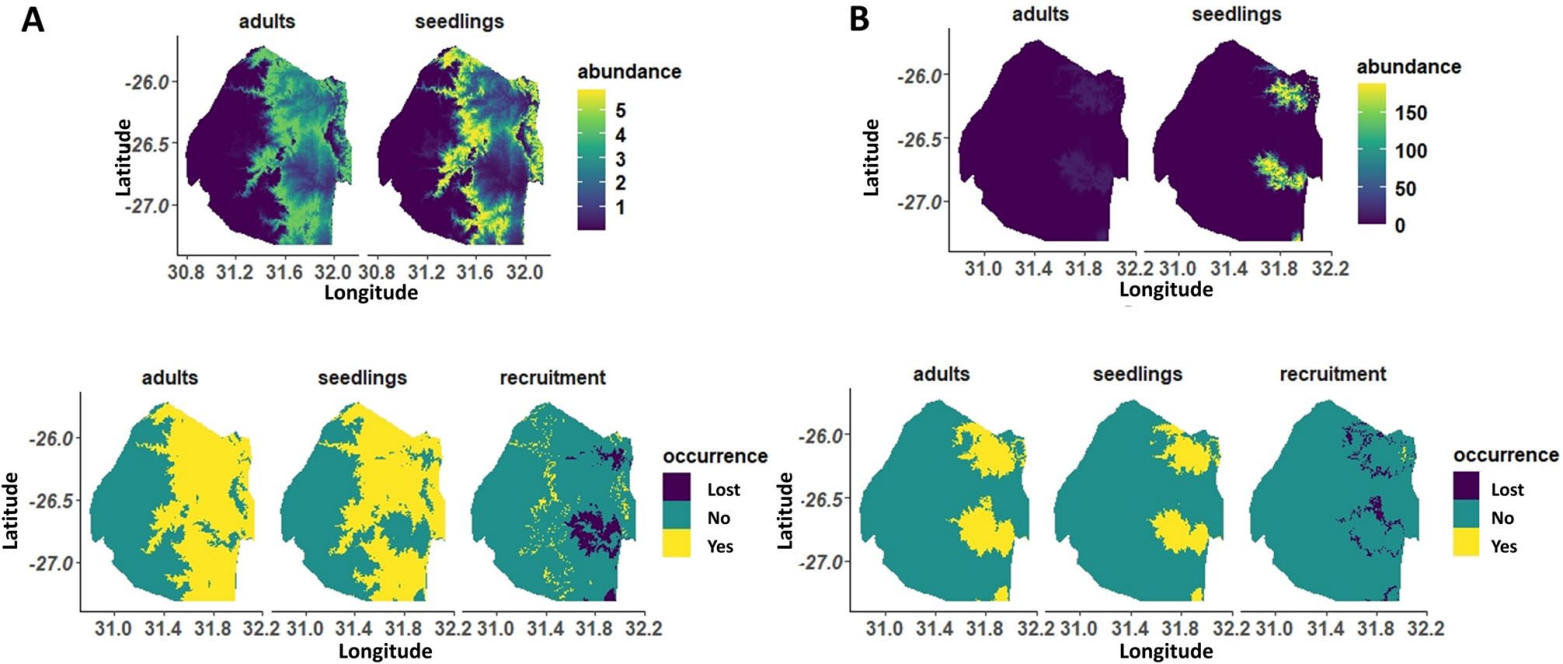
Predicted average abundance (top panel) of marula (**A**) and knobthorn (**B**) across the climate gradient of Eswatini. On the bottom panel, the predicted occurrence of adults, seedlings and recruitment as well as areas where recruitment appears to have been lost (e.g. adults and no seedlings). This figure was produced using the program R^59^.

Based on current regeneration niches projected across Eswatini, we found that predicted range losses were more pronounced for knobthorn than marula (Fig. 3). Marula recovered the same amount of area lost (1197 km^2^) by expanding towards the cooler western region, whereas knobthorn loses 649 km^2^ which is ~ 23% of its initial distribution and this species’ expansion is negligible (< 1% initial distribution) (Table 1).

**Table 1:** Amount of area (km^2^) in Eswatini that gained and lost recruiting marula and knobthorn based on modeled of seedling and adult abundance as a function of 1950–2000 climate data and future projection for 2041–2070 and 2071–2100 under RCPs 4.5 and 8.5.

Species	Recruitment category	1950–2000	RCP 4.5, 2041–2070	RCP 8.5, 2041–2070	RCP 4.5, 2071–2100	RCP 8.5, 2071–2100
Marula	Gained	1197.5	5546.0	5462.6	5276.3	5670.7
	Lost	1197.5	7993.3	7867.7	7801.9	8129.9
Knobthorn	Gained	21.5	1108.6	1117.0	1146.8	1178.3
	Lost	648.5	2333.9	2333.9	2332.4	2332.5

Areas with seedlings but no adults were classified as *Gained*, and areas with adults and no seedling were classified as *Lost*. Initial size of area occupied by marula and knobthorn was 8999.5 and 2854.9 km^2^ respectively.

### Predicting future distributions of abundance

**Figure 4:**
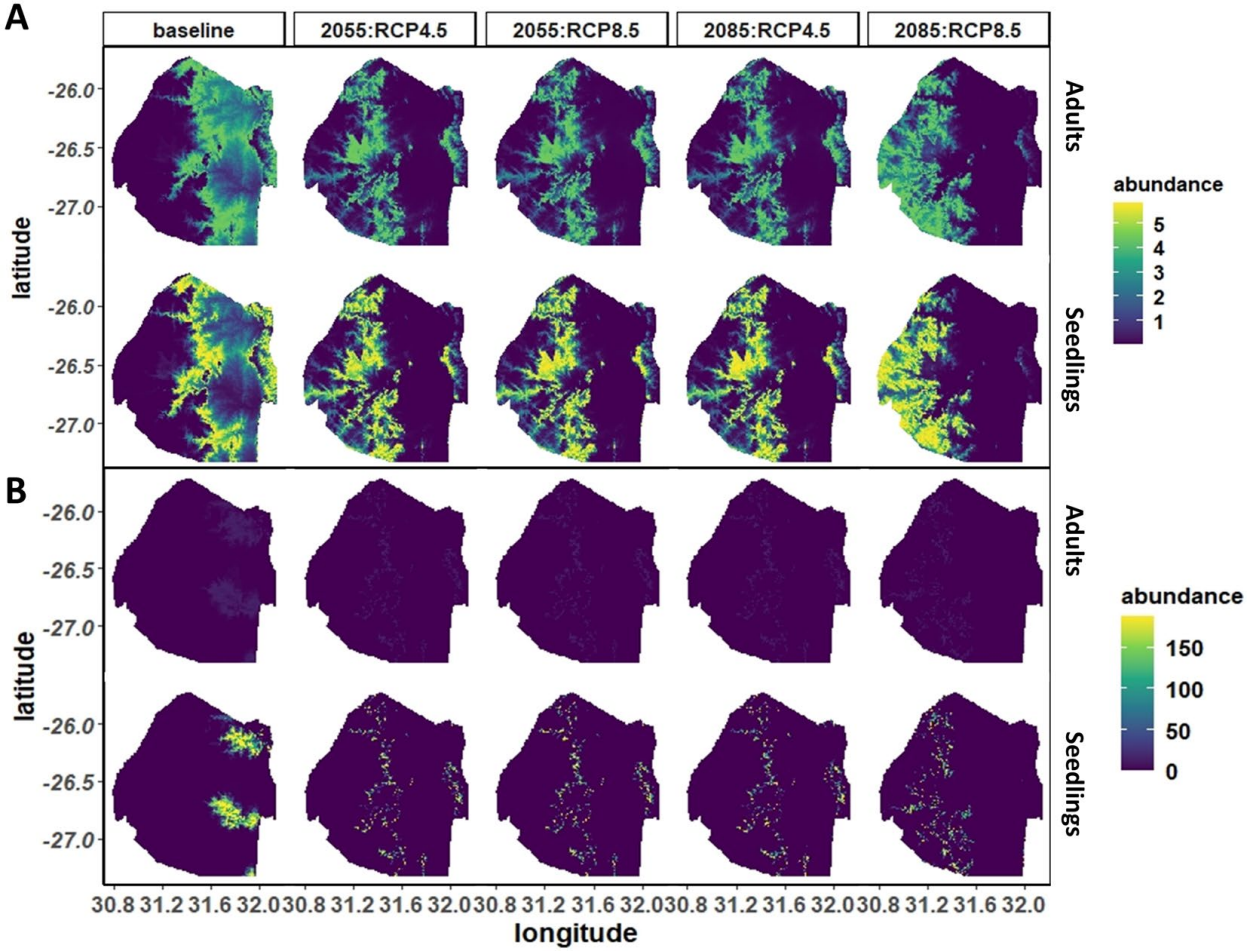
Predicted average abundances of (**A**) marula and (**B**) knobthorn adults and seedlings predicted across the climate gradient of Eswatini (current) and projected for the years 2041–2070 and 2071–2100. Future projections were modeled under the IPCC-AR5 RCP4.5 and RCP8.5. This figure was produced using the program R^59^.

We observed a westward shift towards central Eswatini in both the distributions of marula and knobthorn (Fig. 4) based on projected climate scenarios from 2041 to 2070. The population shifts resulted in both species being extirpated from their current ranges, which are in the east of Eswatini. Remnant populations of marula, however, were projected to survive on the cooler elevated parts of the eastern region, while knobthorn populations started to emerge there. For both marula and knobthorn distributions, predicted shifts for 2041–2070 were similar under both IPCC-AR5 RCP4.5 and RCP8.5 climate projections and persisted to 2071–2100 under RCP4.5 scenario (Fig. 4). Under the RCP8.5 scenario, suitable climate for both species transitioned further west into the grassland biomes and while marula abundance and range distribution were drastically reduced, remnant knobthorn populations were extirpated from the cooler parts of the eastern region by 2071–2100.

Projecting the spatial distribution of marula and knobthorn outside of the range our field work to the broader extent of southern Africa, we found little evidence for future co-occurrences (see Supplementary Fig. S2 online) of knobthorn and marula. Our projections suggest greater potential for marula to track a moving climate, particularly under intermediate (RCP 4.5) emission scenarios. However, there were noticeable contractions in marula distributions in 2071–2100 under PCP8.5. Alternatively, our projections for knobthorn suggested limited distributions centered entirely within and then outside 2041–2070 predicted ranges by 2071–2100 under RCP 4.5 and RCP 8.5 respectively.

### Realism of future distribution of abundance

The expansion beyond current distribution ranges predicted for marula in 2041–2070 appears realistic based on the current regeneration patterns seen in seedling distributions which show regeneration in previously cooler climates (Fig. 3). Thus, projected range expansions outside of the current range of adults was supported by our field study. Similarly, finding few seedlings in marula's core distribution suggests predicted range losses are also probable.

Based on the species’ current regeneration niche, future knobthorn distributions for both 2041–2070 and 2071–2100 cannot be reconciled with the species’ current distributions (Fig. 3). We found no evidence for range expansions or shifts towards newly suitable areas, in fact, contractions were occurring from the outer extent of the species range, further increasing distances between current and expected future distributions (2041–2070 and 2071–2100).

## Discussion

Marula-knobthorn dominated savannas are a hallmark of the savannas of southeastern Africa. Our results suggest that different responses^11^ of these two keystone species to climate change may decouple them^38^ in future savannas. Marula and knobthorn showed idiosyncratic responses across a range of climate conditions, with marula expanding beyond current ranges to make up for lost distributions, while knobthorn patches receded towards the core of their ranges. This suggests that these trees may be losing suitable climate within current ranges and while marula shows evidence of tracking a moving climate, knobthorn is unlikely to do so^11^. In addition, knobthorn’s patchy distributions were limited to a smaller geographic area, such that any range loss may cause local extirpations.

### Knobthorn distribution of abundance

We found knobthorn seedlings and adults occurred within a narrow range (21.5–22.5 °C) of annual mean temperature. Similarly, Smith et al.^38^ found knobthorn to occur within a narrow climate gradient, limited by precipitation (635–900 mm), even though temperature ranges were suitable. Nonetheless, experimentally determined temperature ranges for knobthorn germination were broader^37^ (20.0–43.0 °C) and regeneration appears to be limited by water and not temperature during the growing season^34,37,56^. While our models with precipitation variables did not provide the best explanation of knobthorn distributions, precipitation of the wettest month and PET were positively associated knobthorn abundance, suggesting some rainfall in addition to temperature may also influence on the distribution of knobthorn.

Regardless of the specific climatic influences, this species’ inability to establish outside of its core range suggests other potential limitations (e.g., land use, terrain and soil properties and herbivory) to occupying broader climatic ranges. These barriers will make it unlikely for the species to establish in our predicted future distributions both in Eswatini (Fig. 4) and across southern Africa (see Supplementary Fig. S2 online), which occur respectively tens to hundreds of kilometers away from current distributions. Our observed lack of recruitment in climate suitable areas may also be a function of shade, an important barrier to regeneration of shade-intolerant savanna tree species like knobthorn^74^. Fire suppression, common across our study area, has been shown to filter out shade-intolerant in favor shade tolerant trees^75^. We suspect that knobthorn seedlings are failing to establish in the shady conditions of closed canopies that have been facilitated by a lack of disturbance from fire and large herbivores (e.g., elephants)^12,47^. These disturbances create gaps in the canopy which can trigger regeneration.

### Marula distribution of abundance

Our field data suggests marula populations are contracting at the warmer sites and expanding towards cooler temperatures outside their current adult range. If predicted future distributions in Eswatini, based on current seedling distributions, are consistent over time, marula would be unlikely to persists in some of the regions’ hottest areas, with regional temperature increases projected to be > 4 °C by 2041–2070^76^ and by 2071–2100 for Eswatini^41^. However, the species ability to disperse and recruit in newly suitable climate, beyond current range distributions could counter these effects.

A lack of seedlings does not necessarily equate to local extirpations, but persistence of conditions that result in missing size classes eventually do^45^. From our study, the absence of seedling size classes < 3 m was an indication that the climate has not been suitable for approximately a decade^77^ and there has been no successful episodic recruitment. In most African savannas, missing size classes and absence of marula populations in climatically suitable habitats is a function of interaction of fire and herbivory, particularly where elephants are present^38,45^. However, our study areas did not have elephants and there was only minimal intensive burning^78^, suggesting the absence of juvenile size classes in areas where adult trees persisted was more a function of climatic unsuitability.

Additionally, due to low seedling recruitment, our classification of seedlings (height < 3 m) was very crude and possibly included resprouting individuals that may have already been on the landscape for over a decade^77^. This likely resulted in a conservative estimate on the extent of climatic range loss for future marula and knobthorn distributions. A subset of seedling recruitment that happened only in the last 1–2 years of sampling could show more pronounced changes in optimal temperature differences between seedlings and adults, particularly with the marula populations. Nonetheless, even with these coarse classifications we were able to demonstrate the importance of separating out different size and age class of long-lived trees to get a better mechanistic understanding of how they are likely to respond to our planets rapidly changing climate.

### Modeling considerations

While the patterns in our models were clear, there are number of important considerations in their interpretation. We have less confidence in our projection in southern Africa than we do in our projections within our study areas in Eswatini. Specifically, trees outside of our sampled population may have genetic adaptations that would alter our projections^79^. Another potential source of uncertainly in our models comes from plants response to elevated levels of CO_2_. There is evidence that elevated CO_2_ may influence vegetation dynamics in savannas by enhancing tree growth^80^. While our climate projections accounted for different concentrations of CO_2,_ they did not account for the potentially direct influence of CO_2_ on abundance via carbon fertilization. Accordingly, while the idiosyncratic responses of marula and knobthorn were consistent across our study, there are several uncertainties surrounding our modeled projections.

### Implications for conservation

Our results indicate that the highly specific response of individual species to climate change has the potential to create no-analog terrestrial communities^11^. We found that in coming decades, without human intervention in the form of translocation it is unlikely that populations of knobthorn will establish in areas with marula. On the other hand, range expansion of marula was not limited by the absence of the species’ key disperser, elephants^14^, within the study area. Unlike many tree species whose ability to track climate change has been impeded by defaunation^15^, for marula, human mediated dispersal seems to have effectively replaced elephant dispersal by providing the long-distance dispersal events necessary to track a moving climate^6^.

The idiosyncratic responses to climate change displayed by two co-occurring and dominant species, could result in the creation of novel no-analog communities^81^. This is likely to have direct impacts on the diversity^11^ of communities in the savannas of southeastern Africa. These changes in vegetation composition tend to have cascading effects, rearranging faunal communities^11,81^*.* Additionally, because these tree species are moving at a slower pace than climate change, the expected shifts in mammals^82^ and possibly other terrestrial animals towards cooler climates is unlikely to be matched by shifts in the resources they are provided by savanna trees^14,33,48,83^.

## Supplementary Information

Supplementary Information.

## Data Availability

The datasets generated during and/or analysed during the current study are available from the corresponding author on reasonable request.

## References

[1] Masson-Delmotte, V. *et al.* Climate change 2021: The physical science basis. In *Contribution of working group I to the sixth assessment report of the intergovernmental panel on climate change* **2** (2021).

[2] Sala OE (2000). Biodiversity—Global biodiversity scenarios for the year 2100. Science.

[3] Loarie SR (2009). The velocity of climate change. Nature.

[4] Manning AD, Fischer J, Lindenmayer DB (2006). Scattered trees are keystone structures—Implications for conservation. Biol. Conserv..

[5] Lindenmayer DB (2014). New policies for old trees: Averting a global crisis in a keystone ecological structure. Conserv. Lett.

[6] Corlett RT, Westcott DA (2013). Will plant movements keep up with climate change?. Trends Ecol. Evol..

[7] Di Pasquale G (2020). Coastal pine-oak glacial refugia in the Mediterranean basin: A biogeographic approach based on charcoal analysis and spatial modelling. Forests.

[8] Thurman LL (2020). Persist in place or shift in space? Evaluating the adaptive capacity of species to climate change. Front Ecol. Environ..

[9] Jetz W, Wilcove DS, Dobson AP (2007). Projected impacts of climate and land-use change on the global diversity of birds. Plos Biol..

[10] Di Marco M (2019). Projecting impacts of global climate and land-use scenarios on plant biodiversity using compositional-turnover modelling. Glob. Chang. Biol..

[11] Tomiolo S, Ward D (2018). Species migrations and range shifts: A synthesis of causes and consequences. Perspect. Plant Ecol. Evol. Syst..

[12] Sankaran M (2005). Determinants of woody cover in African savannas. Nature.

[13] Vogel JC, Fuls A (2005). The life-span of leadwood trees. S. Afr. J. Sci..

[14] Helm C, Wilson G, Midgley J, Kruger L, Witkowski ETF (2011). Investigating the vulnerability of an African savanna tree (*Sclerocarya birrea ssp caffra*) to fire and herbivory. Austral Ecol..

[15] Fricke EC, Ordonez A, Rogers HS, Svenning JC (2022). The effects of defaunation on plants' capacity to track climate change. Science.

[16] Staver AC, Botha J, Hedin L (2017). Soils and fire jointly determine vegetation structure in an African savanna. New Phytol..

[17] Staver AC (2018). Prediction and scale in savanna ecosystems. New Phytol..

[18] Guo D, Arnolds JL, Midgley GF, Foden WB (2016). Conservation of quiver trees in Namibia and South Africa under a changing climate. J. Geosci. Environ. Prot..

[19] Foden W (2007). A changing climate is eroding the geographical range of the Namib desert tree aloe through population declines and dispersal lags. Divers. Distrib..

[20] Scheiter S, Higgins SI (2009). Impacts of climate change on the vegetation of Africa: An adaptive dynamic vegetation modelling approach. Glob. Chang. Biol..

[21] Moncrieff GR, Scheiter S, Slingsby JA, Higgins SI (2015). Understanding global change impacts on South African biomes using dynamic vegetation models. S Afr. J. Bot..

[22] Moncrieff GR, Scheiter S, Langan L, Trabucco A, Higgins SI (2016). The future distribution of the savannah biome: Model-based and biogeographic contingency. Philos. Trans. R. Soc. B. Biol. Sci..

[23] Anwar SA, Diallo I (2022). Modelling the tropical african climate using a state-of-the-art coupled regional climate-vegetation model. Clim. Dyn..

[24] Ngarega BK, Masocha VF, Schneider H (2021). Forecasting the effects of bioclimatic characteristics and climate change on the potential distribution of Colophospermum mopane in southern Africa using maximum entropy (Maxent). Ecol. Inform..

[25] Purves D, Pacala S (2008). Predictive models of forest dynamics. Science.

[26] Guisan A, Thuiller W (2005). Predicting species distribution: Offering more than simple habitat models. Ecol. Lett..

[27] Dyderski MK, Paz S, Frelich LE, Jagodzinski AM (2018). How much does climate change threaten European forest tree species distributions?. Glob. Chang. Biol..

[28] Getzin S, Wiegand T, Wiegand K, He FL (2008). Heterogeneity influences spatial patterns and demographics in forest stands. J. Ecol..

[29] Grubb PJ (1977). The maintenance of species-richness in plant communities: The importance of the regeneration niche. Biol. Rev..

[30] Poorter L (2007). Are species adapted to their regeneration niche, adult niche, or both?. Am. Nat..

[31] Tiscar PA (2017). Regeneration of three pine species in a Mediterranean forest: A study to test predictions from species distribution models under changing climates. Sci. Total Environ..

[32] Stevens N, Archibald SA, Bond WJ (2018). Transplant experiments point to fire regime as limiting savanna tree distribution. Front. Ecol. Evol..

[33] Helm CV, Witkowski ETF, Kruger L, Hofmeyr M, Owen-Smith N (2009). Mortality and utilisation of Sclerocarya birrea subsp Caffra between 2001 and 2008 in the Kruger National Park. South Africa. S. Afr. J. Bot..

[34] Massad TJ, Castigo T (2016). Investigating possible effects of climate change on tree recruitment: Responses of abundant species to water stress in Gorongosa national park. S. Afr. J. Bot..

[35] Breshears DD, Huxman TE, Adams HD, Zou CB, Davison JE (2008). Vegetation synchronously leans upslope as climate warms. Proc. Natl. Acad. Sci. USA.

[36] Midgley JJ, Bond WJ (2001). A synthesis of the demography of African acacias. J. Trop. Ecol..

[37] Stevens N, Seal CE, Archibald S, Bond W (2014). Increasing temperatures can improve seedling establishment in arid-adapted savanna trees. Oecologia.

[38] Smith A, Page B, Duffy K, Slotow R (2012). Using maximum entropy modeling to predict the potential distributions of large trees for conservation planning. Ecosphere.

[39] Shackleton SE (2002). Use patterns and value of savanna resources in three rural villages in South Africa. Econ. Bot..

[40] Dai AG (2013). Increasing drought under global warming in observations and models. Nat. Clim. Chang..

[41] Dlamini W (2011). Probabilistic spatio-temporal assessment of vegetation vulnerability to climate change in Swaziland. Glob. Chang. Biol..

[42] Remmelzwaal, A. & Vilakati, J. Physiographic map of Swaziland. *AG: SW89/001 Field Document* **4** (1993).

[43] Tews J (2004). Animal species diversity driven by habitat heterogeneity/diversity: The importance of keystone structures. J. Biogeogr..

[44] Trollope W, van Wilgen B, Trollope LA, Govender N, Potgieter AL (2014). The long-term effect of fire and grazing by wildlife on range condition in moist and arid savannas in the Kruger national park. Afr. J. Range Forage Sci..

[45] Helm CV, Witkowski ETF (2012). Characterising wide spatial variation in population size structure of a keystone African savanna tree. For. Ecol. Manag..

[46] Manning AD, Fischer J, Lindenmayer DB (2006). Scattered trees are keystone structures–implications for conservation. Biol. Conserv..

[47] McCleery R (2018). Animal diversity declines with broad-scale homogenization of canopy cover in African savannas. Biol. Conserv..

[48] Fornara DA, Du Toit JT (2007). Browsing lawns? Responses of acacia nigrescens to ungulate browsing in an African savanna. Ecology.

[49] Boundja RP, Midgley JJ (2010). Patterns of elephant impact on woody plants in the Hluhluwe-Imfolozi park, Kwazulu-Natal, South Africa. Afr. J. Ecol..

[50] Higgins SI, Shackleton CM, Robinson ER (1999). Changes in woody community structure and composition under constrasting landuse systems in a semi-arid savanna, South Africa. J. Biogeogr..

[51] Helm CV, Scott SL, Witkowski ETF (2011). Reproductive potential and seed fate of Sclerocarya birrea subsp caffra (marula) in the low altitude savannas of South Africa. S. Afr. J. Bot..

[52] Mduma SAR, Sinclair ARE, Turkington R (2007). The role of rainfall and predators in determining synchrony in reproduction of savanna trees in Serengeti National Park, Tanzania. J. Ecol..

[53] Palmer, E. & Pitman, N. *Trees of Southern Africa: Covering all known indigenous species in the Republic of South Africa, South-West Africa, Botswana, Lesotho & Swaziland. Vol 1 & 2.* A. A. Balkema (1972).

[54] Midgley JJ, Gallaher K, Kruger LM (2012). The role of the elephant (Loxodonta africana) and the tree squirrel (Paraxerus cepapi) in marula (Sclerocarya birrea) seed predation, dispersal and germination. J. Trop. Ecol..

[55] Muller K, O'Connor TG, Henschel JR (2016). Impact of a severe frost event in 2014 on woody vegetation within the Nama-Karoo and semi-arid savanna biomes of South Africa. J. Arid Environ..

[56] O'Keefe K, Nippert JB, Swemmer AM (2016). Savanna tree seedlings are physiologically tolerant to nighttime freeze events. Front. Plant Sci..

[57] Olson DM (2001). Terrestrial ecoregions of the worlds: A new map of life on earth. Bioscience.

[58] Leutner, B., Horning, N. & Leutner, M. B. Package ‘RStoolbox’. *R Foundation for Statistical Computing, Version 0.1* (2017).

[59] R Core Team. in *R: A language and environment for statistical computing* (2012).

[60] Vogel SM (2014). Elephant (Loxodonta africana) impact on trees used by nesting vultures and raptors in South Africa. Afr. J. Ecol..

[61] Lenoir J, Gégout JC, Pierrat JC, Bontemps JD, Dhôte JF (2009). Differences between tree species seedling and adult altitudinal distribution in mountain forests during the recent warm period (1986–2006). Ecography.

[62] Canham CD, Murphy L (2016). The demography of tree species response to climate: Seedling recruitment and survival. Ecosphere.

[63] Brooks ME (2017). glmmTMB balances speed and flexibility among packages for zero-inflated generalized linear mixed modeling. R J..

[64] Zhang H, Yan C, Wu S, Si J, Yi X, Li H, Zhang Z (2021). Effects of masting on seedling establishment of a rodent-dispersed tree species in a warm-temperate region, northern China. Integr. Zool..

[65] Liu Y, Fang S, Chesson P, He F (2015). The effect of soil-borne pathogens depends on the abundance of host tree species. Nat. Commun..

[66] Fick SE, Decker C, Duniway MC, Miller ME (2016). Small-scale barriers mitigate desertification processes and enhance plant recruitment in a degraded semiarid grassland. Ecosphere.

[67] Bolker, B. & R Core Team. Package ‘bbmle’. *Tools Gen. Maximum Likelihood Estim.* (2017).

[68] Lüdecke, D. & Lüdecke, M. D. Package ‘sjstats’. *Statistical functions for Regression Models, Version 0.17.*5 (2019).

[69] Bjornstad ON, Falck W (2001). Nonparametric spatial covariance functions: Estimation and testing. Environ. Ecol. Stat..

[70] Beale CM, Lennon JJ, Yearsley JM, Brewer MJ, Elston DA (2010). Regression analysis of spatial data. Ecol. Lett..

[71] Hijmans RJ (2015). Package ‘raster’. R Package.

[72] Ali H (2021). Expanding or shrinking? Range shifts in wild ungulates under climate change in Pamir-Karakoram mountains, Pakistan. PLoS ONE.

[73] Platts PJ, Omeny PA, Marchant R (2015). AFRICLIM: High-resolution climate projections for ecological applications in Africa. Afr. J. Ecol..

[74] Pausas JG, Bond WJ (2021). Alternative biome states challenge the modelling of species' niche shifts under climate change. J. Ecol..

[75] Abreu RCR (2017). The biodiversity cost of carbon sequestration in tropical savanna. Sci. Adv..

[76] Hulme M, Doherty R, Ngara T, New M, Lister D (2001). African climate change: 1900–2100. Clim. Res..

[77] Wakeling JL, Staver AC, Bond WJ (2011). Simply the best: The transition of savanna saplings to trees. Oikos.

[78] Dlamini WM (2010). A Bayesian belief network analysis of factors influencing wildfire occurrence in Swaziland. Environ. Model. Softw..

[79] Alberto FJ (2013). Potential for evolutionary responses to climate change–evidence from tree populations. Glob. Chang. Biol..

[80] Midgley GF, Bond WJ (2015). Future of African terrestrial biodiversity and ecosystems under anthropogenic climate change. Nat. Clim. Chang..

[81] Parr CL, Gray EF, Bond WJ (2012). Cascading biodiversity and functional consequences of a global change-induced biome switch. Divers. Distrib..

[82] Thuiller W (2006). Vulnerability of African mammals to anthropogenic climate change under conservative land transformation assumptions. Glob. Chang. Biol..

[83] Monadjem A, Raabe T, Dickerson B, Silvy N, McCleery RA (2010). Roost use by two sympatric species of Scotophilus in a natural environment. S. Afr. J. Wildl. Res..

