# Supplementary material for: Climate change decouples dominant tree species in African savannas
Source: Sci Rep. 2023 May 10;13:7619. doi: 10.1038/s41598-023-34550-9 (PMC10172338; doi:10.1038/s41598-023-34550-9)

**Supplemental Table Legends**

**Table 1**: WorldClim bioclimatic variables derived from monthly temperature and rainfall values.

**Table 2.** Models of climate variables and distributions of marula (a) and knobthorn (b) trees in Eswatini.

**Table 3**. Estimates and 95% CI for the best models of marula (a) and knobthorn (b).

Area covered where species distributions were either present, absent or lost as indicated by the

**Supplemental Table S1**

| Model name | Description | known knobthorn range | known marula range | Sampled ranges | Annuclim code |
| --- | --- | --- | --- | --- | --- |
| temp | Mean Annual Temperature | 20.9 – 24.6 º C | 19.0 – 26 º C | 15.6 - 22.7 º C | Bioclim01 |
| maxtemp | Maximum Temperature of warmest month | 32.4 – 38.1 º C | 27.0 – 35.0 º C | 23.8 – 31.9 º C | Boiclim05 |
| frost | Minimum Temperature of coldest month | 7.4 – 10.3 º C | 5.0 -17.5 º C | 4.3 – 11.1 º C | Bioclim06 |
| prec | Annual Precipitation | 344 – 707 mm | 500 – 1600 mm | 544 – 1280 mm | Bioclim12 |
| maxprec | Precipitation of wettest month | NA | NA | 88 – 227 mm | Bioclim13 |
| pet | Potential evapotranspiration |  | 1100 – 1700 mm | 1309 – 1606 mm | PET |

Values for the complete climate range of marula in southern Africa have been extracted from Hall and others 2002. Reported values for knobthorn climate ranges are based on distributions in South Africa (Stevens and others 2018), which represents the arid bounds for the species.

| Supplemental Table S2 | | | | | | | | | |  |
| --- | --- | --- | --- | --- | --- | --- | --- | --- | --- | --- |
| Model name | Total Abundance | | | Seedlings | | | Adults | | |  |
|  | ΔAIC | R^2^_cond._ | R^2^_marg._ | ΔAIC | R^2^_cond._ | R^2^_marg._ | ΔAIC | R^2^_cond._ | R^2^_marg._ | df |
| A) Marula |  |  |  |  |  |  |  |  |  |  |
| maxtemp + maxtemp^2^ | 0.0 | 0.86 | 0.81 | 0.0 | 0.82 | 0.78 | 0.0 | 0.85 | 0.80 | 6 |
| temp + temp^2^ | 0.6 | 0.87 | 0.81 | 0.0 | 0.84 | 0.81 | 0.6 | 0.85 | 0.80 | 6 |
| pet + pet^2^ | 1.3 | 0.67 | 0.56 | 2.4 | 0.50 | 0.40 | 0.8 | 0.67 | 0.56 | 6 |
| temp | 6.8 | 0.60 | 0.34 | 9.2 | 0.44 | 0.11 | 6.7 | 0.85 | 0.35 | 5 |
| maxtemp | 7.8 | 0.58 | 0.31 | 9.5 | 0.44 | 0.10 | 7.8 | 0.57 | 0.32 | 5 |
| pet | 8.1 | 0.58 | 0.31 | 9.4 | 0.42 | 0.10 | 7.0 | 0.56 | 0.32 | 5 |
| mintemp + mintemp^2^ | 9.4 | 0.70 | 0.51 | 5.5 | 0.52 | 0.38 | 9.2 | 0.70 | 0.51 | 6 |
| maxprec + maxprec^2^ | 15 | 0.58 | 0.22 | 12.1 | 0.40 | 0.06 | 14.0 | 0.62 | 0.42 | 6 |
| mintemp | 15 | 0.58 | 0.21 | 11.5 | 0.42 | 0.45 | 14.3 | 0.57 | 0.22 | 5 |
| prec + prec^2^ | 16.2 | 0.56 | 0.29 | 12.9 | 0.39 | 0.07 | 15.2 | 0.55 | 0.30 | 6 |
| prec | 17.5 | 0.55 | 0.18 | 11.9 | 0.41 | 0.04 | 16.8 | 0.54 | 0.18 | 5 |
| maxprec | 18.6 | 0.56 | 0.17 | 11.3 | 0.40 | 0.06 | 18.1 | 0.54 | 0.17 | 5 |
| null | 25.1 | 0.54 | 0.00 | 11.5 | 0.41 | 0.00 | 25.0 | 0.54 | 0.00 | 4 |
| B) Knobthorn |  |  |  |  |  |  |  |  |  |  |
| temp + temp^2^ | 0.0 | 1.00 | 1.00 | 0.0 | 1.00 | 1.00 | 0.0 | 1.00 | 1.00 | 6 |
| pet | 4.6 | 0.86 | 0.85 | 7.8 | 0.89 | 0.87 | 2.2 | 0.84 | 0.83 | 6 |
| pet + pet^2^ | 5.8 | 1.00 | 1.00 | NA | 1.00 | 1.00 | 3.4 | 1.00 | 1.00 | 6 |
| maxtemp + maxtemp^2^ | 8.8 | 1.00 | 1.00 | NA | 1.00 | 1.00 | 9.4 | 1.00 | 1.00 | 6 |
| prec + prec^2^ | 9.9 | 0.98 | 0.97 | 13.8 | 0.97 | 0.96 | 4.8 | 0.97 | 0.96 | 6 |
| maxprec + maxprec^2^ | 13.7 | 0.97 | 0.96 | NA | NA | NA | 11.0 | 0.96 | 0.96 | 6 |
| prec | 13.8 | 0.63 | 0.51 | 15.4 | NA | NA | 10.9 | 0.57 | 0.48 | 5 |
| maxptemp | 14.5 | 0.71 | 0.63 | 13.9 | 0.80 | 0.74 | 14.3 | 0.67 | 0.59 | 5 |
| temp | 15.1 | 0.71 | 0.62 | 10.4 | 0.75 | 0.67 | 13.1 | 0.67 | 0.58 | 5 |
| mintemp + mintemp^2^ | 16.7 | 0.94 | 0.93 | NA | NA | NA | 18.3 | 0.93 | 0.92 | 6 |
| maxprec | 17.8 | 0.61 | 0.43 | 17.4 | NA | NA | 15.1 | 0.55 | 0.40 | 5 |
| mintemp | 22.6 | 0.57 | 0.23 | NA | 0.57 | 0.51 | NA | 0.56 | 0.23 | 5 |
| null | 26.2 | 0.77 | 0.00 | NA | 0.76 | 0.00 | 20.2 | 0.77 | 0.00 | 4 |

NA ΔAIC values = did not converge. R^2^_cond._ and R^2^_marg._ are the conditional and marginal R^2^ values.

**Supplemental Table S3**

| A) Marula |  | term | β estimate | p-value |
| --- | --- | --- | --- | --- |
| Total Abundance | Annual temp. | *x* | 4.84 (8.72, 0.96) | 0.01 |
|  |  | *x^2^* | -3.31 (-0.30, -6.33) | 0.03 |
|  | Max temp. | *x* | 4.90 (8.45, 1.35) | <0.01 |
|  |  | *x^2^* | -3.73 (-0.70, -6.75) | <0.01 |
|  | PET | *x* | 2.76 (4.31, 1.21) | <0.01 |
|  |  | *x^2^* | -2.37 (-0.77, -3.96) | <0.01 |
| Seedlings | Annual temp | *x* | 3.83 (7.77, -1.06) | 0.06 |
|  |  | *x^2^* | -3.90 (-0.58, -7.22) | 0.02 |
|  | max temp | *x* | 3.84 (7.51, 1.77) | 0.04 |
|  |  | *x^2^* | -4.00 (-0.74, -7.26) | 0.02 |
|  | PET | *x* | 1.57 (2.98, 1.61) | 0.03 |
|  |  | *x^2^* | -2.40 (-0.77, -4.03) | <0.01 |
| Adults | Annual temp | *x* | 4.56 (8.24, 0.88) | 0.02 |
|  |  | *x^2^* | -3.00 (-0.14, -5.86) | 0.04 |
|  | Max temp | *x* | 4.64 (8.03, 1.26) | <0.01 |
|  |  | *x^2^* | -3.43 (-0.55, -6.31) | 0.02 |
|  | PET | *x* | 2.73 (4.27, 1.20) | <0.01 |
|  |  | *x^2^* | -2.17 (-0.63, -3.71) | <0.01 |
| B) Knobthorn |  |  |  |  |
| Total Abundance | Annual temp. | *x* | 261.46 (455.36, 67.57) | <0.01 |
|  |  | *x^2^* | -111.65 (-29.37, -193.93) | <0.01 |
| Seedlings | Annual temp | *x* | 454.17 (805.76,102.57) | 0.01 |
|  |  | *x^2^* | -186.19 (-41.69, -330.69) | 0.01 |
| Adults | Annual temp | *x* | 247.90 (484.9, 10.87) | 0.04 |
|  |  | *x^2^* | -105.05 (-5.84, -204.26) | 0.04 |

**Supplemental Figure Legends**

**Supplemental** **Fig. S1:** Predicted abundance for total, seedlings and adult knobthorn across a gradient of Annual mean temperature (ºC).

**Supplemental Fig. S2**: Regional projections of marula and knobthorn adults and seedlngs in southern African savannas for the years 2055 and 2085 under the IPCC-AR5 RCP4.5 and RCP8.5.

**Supplemental Figure 1**


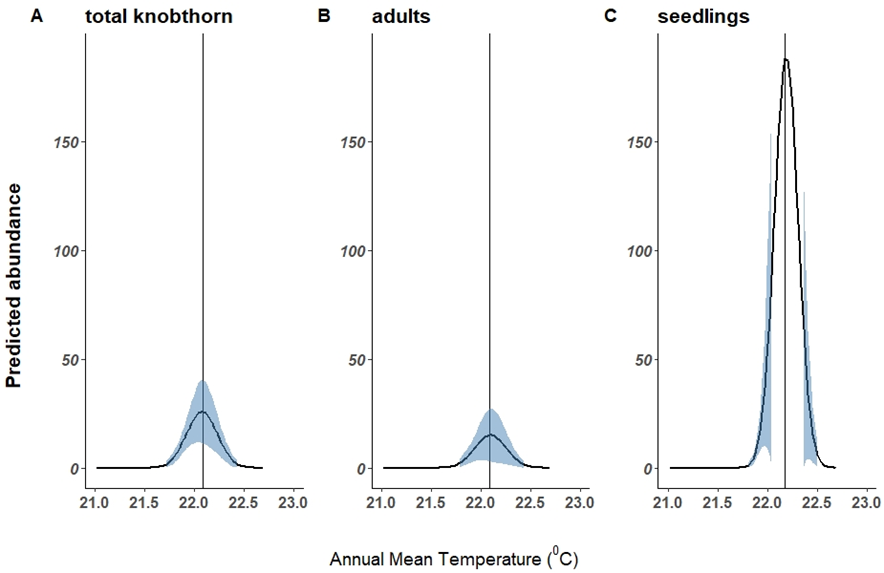


**Supplemental figure 2**


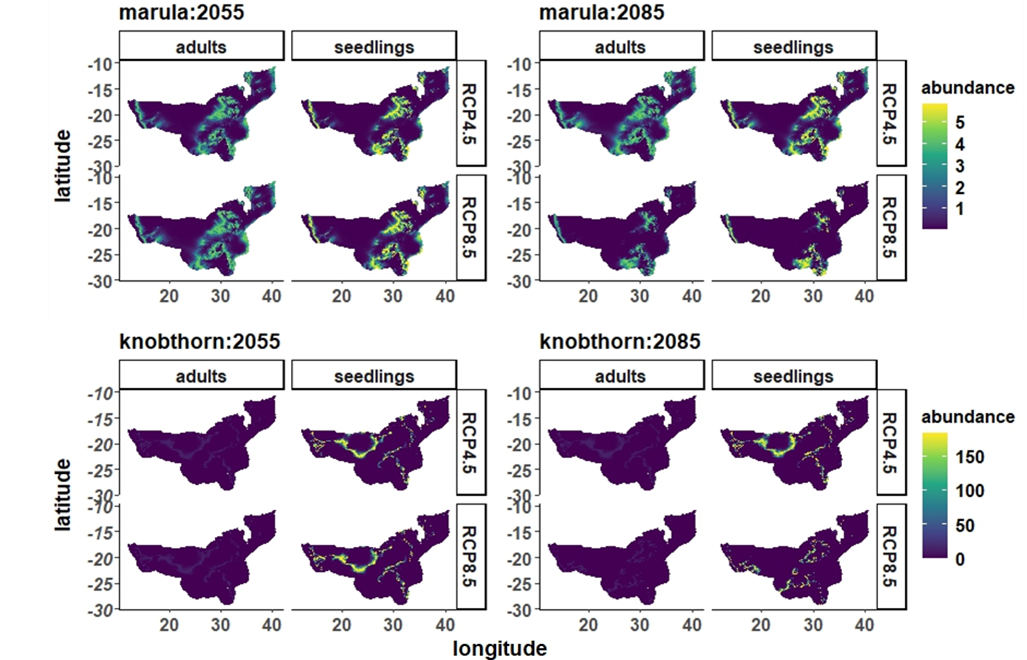

Supplement: Supplementary file 1 — Supplementary Information. [file 41598_2023_34550_MOESM1_ESM.docx]
